# Development of a precision medicine pipeline to identify personalized treatments for colorectal cancer

**DOI:** 10.1186/s12885-020-07090-y

**Published:** 2020-06-24

**Authors:** Erdem Altunel, Roham S. Roghani, Kai-Yuan Chen, So Young Kim, Shannon McCall, Kathryn E. Ware, Xiling Shen, Jason A. Somarelli, David S. Hsu

**Affiliations:** 1 Department of Medicine, Division of Medical Oncology, Duke University Medical Center, 3008 Snyderman Building, 905 S. LaSalle St., Durham, NC 27710 USA; 2 Center for Genomics and Computational Biology, Duke University, Durham, North Carolina USA; 3 Department of Biomedical Engineering, Duke University, Durham, North Carolina USA; 4 Duke Functional Genomics Core, Duke University, Durham, North Carolina USA; 5 Department of Pathology, Duke University, Durham, North Carolina USA

**Keywords:** Metastatic colorectal cancer, Patient derived xenograft, High-throughput drug screen, Ponatinib

## Abstract

**Background:**

Metastatic colorectal cancer (CRC) continues to be a major health problem, and current treatments are primarily for disease control and palliation of symptoms. In this study, we developed a precision medicine strategy to discover novel therapeutics for patients with CRC.

**Methods:**

Six matched low-passage cell lines and patient-derived xenografts (PDX) were established from CRC patients undergoing resection of their cancer. High-throughput drug screens using a 119 FDA-approved oncology drug library were performed on these cell lines, which were then validated in vivo in matched PDXs. RNA-Seq analysis was then performed to identify predictors of response.

**Results:**

Our study revealed marked differences in response to standard-of-care agents across patients and pinpointed druggable pathways to treat CRC. Among these pathways co-targeting of fibroblast growth factor receptor (FGFR), SRC, platelet derived growth factor receptor (PDGFR), or vascular endothelial growth factor receptor (VEGFR) signaling was found to be an effective strategy. Molecular analyses revealed potential predictors of response to these druggable pathways.

**Conclusions:**

Our data suggests that the use of matched low-passage cell lines and PDXs is a promising strategy to identify new therapies and pathways to treat metastatic CRC.

## Background

Colorectal cancer (CRC) continues to be a major public health problem, both in the United States and worldwide; it is the third most common cancer in the United States with approximately 150,000 new cases per year [1, 2]. Metastatic disease currently remains predominantly incurable, and treatment is primarily for palliation of symptoms and disease control. In general, 5-fluorouracil (5-FU)-containing regimens have formed the backbone of chemotherapy to treat CRC for the last several decades. Recently, additional compounds have proven to be effective as treatment in first, second, and third line metastatic disease. These include both traditional chemotherapeutic agents along with targeted biologic agents [1–3]. Although there have been great strides made to improve the survival of patients with metastatic CRC, the median survival for patients still remains at a mere 30 months [1–3].

Over the past decade, targeting molecular pathways of tumor growth/proliferation has become a major focus of anti-cancer treatments to develop new and novel drugs in CRC. For example, agents like bevacizumab, which targets the vascular endothelial growth factor (VEGF) pathway, or cetuximab and panitumumab, which target the epidermal growth factor receptor (EGFR) pathway, have become standard-of-care therapies. However, once patients have completed treatment or become resistant to these currently-available treatments, there are no effective options left for patients. Unfortunately, new drugs for the treatment of metastatic CRC have been limited, and over the past few years, only two drugs, regorafenib and lonsurf, have been approved in the refractory setting for the treatment of metastatic CRC.

Like most other cancers, the failure rate for new cancer drugs is more than 80% in Phase II and 50% in Phase III [4, 5], and failure rates for both Phase II and Phase III oncology clinical trials have been rising since 2001. Part of the high failure rate results from a relative lack of models that faithfully recapitulate the disease state. To address this lack of models, researchers have turned to patient-derived models of cancer, such as cell lines, organoids, and patient-derived xenografts (PDXs), which are increasingly being accepted as “standard” preclinical models to facilitate the identification and development of new therapeutics. For example, large-scale drug screens of cancer cell line panels have been used to identify sensitivity to a large number of potential therapeutics [6]. Similarly, tumor organoid cultures from CRC specimens have also been used to perform drug screens [7], and PDXs of CRC are also being used to predict drug response [8] and to identify novel drug combinations [9]. Finally, combinations of patient-derived models are currently being explored to develop precision medicine strategies for cancer care [10].

In the current study, we developed a precision medicine strategy for patients with metastatic CRC. Specifically, we developed a series of patient-matched cell lines and PDXs. The cell lines were first used to perform high throughput drug screens to identify potential therapeutic targets, and the matched PDXs were then used to validate these findings. Using this approach, we observed patient-specific heterogeneity in response to both standard-of-care agents and targeted therapies. Among the targeted therapies, ponatinib and trametinib were the most efficacious for different patient-derived models. Further mechanistic studies of ponatinib’s downstream targets demonstrated potential antitumor activity by co-targeting the fibroblast growth factor receptor (FGFR), SRC, platelet derived growth factor receptor (PDGFR) or vascular endothelial growth factor receptor (VEGFR) signaling. Consistent with these observations mining of next-generation RNA sequencing (RNA-Seq) data identified mutations in these pathways as potential molecular predictors of response. Together, our results support the use of a precision medicine pipeline to identify personalized therapies and predictive biomarkers for the treatment of metastatic CRC.

## Methods

### Generation of patient-derived Xenograft models and matched PDX cell lines

Patient derived CRC tumor tissue samples were collected under a Duke Institutional Review Board (IRB) approved protocol (Pro00002435). All participants provided written informed consent to participate in the study. PDX models of CRC were then generated as described previously [11, 12], and all in vivo mouse experiments were performed in accordance with the animal guidelines and with the approval of the Institutional Animal Care and Use committee (IACUC) at the Duke University Medical Center. Briefly, to generate PDXs, tissue samples were washed in phosphate buffered saline (PBS), dissected into small pieces (< 2 mm), and injected into the flanks of 8–10-week-old JAX NOD.CB17-PrkdcSCID-J mice. Mice were purchased from the Duke University Rodent Genetic and Breeding Core and housed in IVC cages containing corn cob using the day to night pattern (7 am- 7 pm) lightening control.

Matched PDX cell lines were generated from the PDXs as follows. Once the PDX tumors reached a size of > 1000 mm3, tumors were harvested, homogenized and grown in 10 cm2 tissue culture-treated dishes in cell culture media (DMEM media, 10% fetal bovine serum (FBS), 10 U/ml penicillin and streptomycin) at 37 °C and 5% CO2. Clonal populations of each cell line were then obtained by isolating a single clone using trypsinization of the clone sealed off from the dish by an O ring. The following matched CRC PDXs and cell lines were generated and used in this study; CRC119, CRC057, CRC240, CRC247, 16–159 and 15–496. Cell lines were authenticated using the Duke University DNA Analysis Facility Human cell line authentication (CLA) service by analyzing DNA samples from each individual cell line for polymorphic short tandem repeat (STR) markers using the GenePrint 10 kit from Promega (Madison, WI, USA).

### High-throughput screening

Automated liquid handling was provided by the Echo Acoustic Dispenser (Labcyte) for drug addition or Well mate (Thermo Fisher) for cell plating, and assays were performed using a Clarioscan plate reader (BMG Labtech). Immediately prior to cell plating, 384 well plates were stamped with 119 FDA-approved drug compounds at a final concentration of 1 uM. The compound library (Approved Oncology Set VI) was provided by the NCI Developmental Therapeutics Program (https://dtp.cancer.gov/). CRC119, CRC057, CRC240, CRC247, 16–159 and 15–496 cell lines were plated in these drug pre-coated plates in a range of 500 and 1000 cells/well. Cell viabilities were assessed via CellTiter-Glo Luminescent Cell Viability Assay (Promega, USA) 72 h after cell plating. Percent killing was quantified using the following formula: 100*[1-(the value average CellTiterGlo^drug^/average CellTiterGlo^DMSO^)].

### In vitro drug sensitivity assays

CRC119, CRC057 and CRC240 cell lines were cultured in DMEM + 10% FBS + 1% Penicillin/Streptomycin and plated in drug-free medium at concentrations between 3000 and 6000 cells/well in 96 well plate. Ponatinib (AP24534) was purchased from Selleck Chemicals (Houston, TX) and was solubilized in DMSO to a final concentration of 50 mM. Five replicates were used for each drug concentration. Each cell line was exposed to a series of seven different drug concentration (1.6 nM – 25 μM) after 24 h of incubation at 37 °C. Cell viability was measured 72 h following the addition of DMSO or drug via CellTiter-Glo Luminescent Cell Viability Assays (Promega, USA), and IC50 values were calculated for each cell line using GraphPad Prism software (La Jolla, CA, USA).

### In vivo drug sensitivity assays

To test the sensitivity of CRC119, CRC057 and CRC240 PDXs to ponatinib, oxaliplatin and irinotecan, 150 μl of homogenized PDX tissue-PBS suspensions at 150 mg/ml concentration were subcutaneously injected into the right flanks of 5 female and 5 male mice (JAX NOD.CB17- PrkdcSCID-J, 10 weeks old, ~ 25 g). Following injection, mice were randomized into control and treatment groups. 5 times a week in the morning oral dosing of ponatinib (30 mg/kg); 5 times a week in the morning intraperitoneal dosing of oxaliplatin (10 mg/kg) and irinotecan (10 mg/kg) were initiated when tumor volumes reached approximately 150 mm^3^. Tumor volume measurements were performed every other day using calipers, and the following formula was used to calculate tumor size: (length x (width)2)/2. The mice were euthanized by bilateral thoracotomy under CO_2_ induced anesthesia at the end of the study.

### Western blotting analysis

Western blots were performed pre- and 24-h post-treatment with vehicle (DMSO) or ponatinib at IC50 doses of each cell line. A total of 100,000 cells were lysed in radioimmunoprecipitation assay (RIPA) lysis buffer supplemented with protease and phosphatase inhibitor cocktail (company), and a total of 50 μg of RIPA lysate was electrophoretically separated at 200 V on 4–20% sodium dodecyl sulfate polyacrylamide gels using a BioRad MiniProtean Tetra system. Subsequent to transfer onto nitrocellulose membranes at 50 V for 2 h, membranes were blocked in StartingBlock T20 (ThermoFisher) for 1 h at room temperature, followed by incubation in primary antibody diluted in StartingBlock T20 overnight at 4 °C with rocking. Membranes were washed three times for 5 min each in PBS + 0.05% Tween-20, incubated in corresponding Horse Radish Peroxidase (HRP) conjugated secondary antibodies according to the specifications of the manufacturer’s protocols. The Odyssey Infrared Imaging System (LI-COR Biosciences) was used for membrane imaging. The following primary antibodies and dilutions were used: FGFR1 (#9740), FGFR2 (#11835), pFGFR (#3471), pSRC (#2105), pVEGFR (#12599), pERK (#4377), pAkt (#4060), pSTAT5 (#4322), pSTAT3(#9131), β-Actin (#4970) antibodies (Cell Signaling Technology Inc., USA); pPDGFR (ab5460) antibody (Abcam, Cambridge, MA, USA); and pABL (sc-293,130), FGFR3 (#sc-390,423), FGFR4 (#sc-136,988) (Santa Cruz Biotechnology, Santa Cruz, CA, USA). All antibodies were used at 1:1000 dilutions.

### Data analysis and statistics

GraphPad Prism 6 software (La Jolla, CA, USA) was used for in vitro and in vivo data recording and statistical analysis. 2-way ANOVA analysis was used to compare the tumor size between control groups and treatment groups in vivo and drug sensitivity in vitro. A *p*-value < 0.05 was considered statistically significant.

### RNA-Seq analysis

The RNA-seq libraries were prepared and sequenced in Illumina HiSeq 4000 with 150 bp paired-end reads. The reads were aligned to human genome hg19. In variant calling analysis, pipeline of GATK [13] developed by Broad Institute is followed (https://software.broadinstitute.org/gatk/). One hundred fifty bp PE reads were first aligned using STAR-2pass method with default parameters. The output SAM files were processed by using Picard (http://broadinstitute.github.io/picard/) subsequently to add read group, sort, mark duplicates and index. GATK tool was used for variant calling, and SnpEff was used to annotated the identified variants. In fusion analysis, STAR-Fusion (https://www.biorxiv.org/content/early/2017/03/24/120295) package developed by the Broad Institute was applied to detect fusion reads in the paired-end RNA-seq data with default parameters.

## Results

### Development of preclinical models for a precision medicine pipeline

**Fig. 1:**
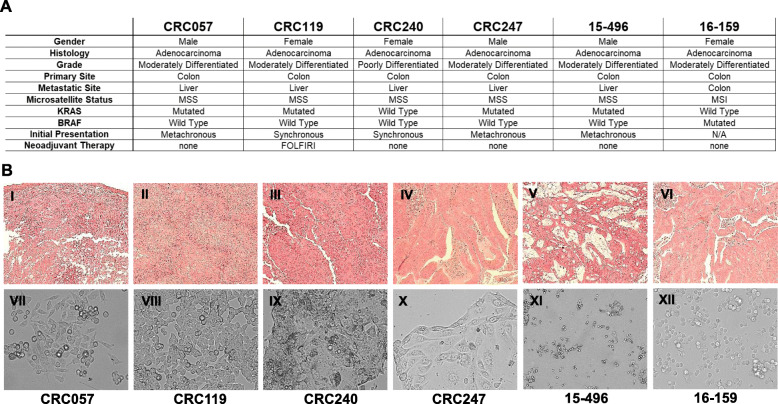
Development of patient-derived models of cancer to identify new treatments for metastatic colorectal cancer. **a.** Clinical characteristics of the six metastatic colorectal cancer patients used to generate the patient-derived models. Tumor tissues of CRC057, CRC119, CRC240, CRC247 and 15–496 were obtained from the liver metastasis; the tissue of 16–159 was obtained from a primary tumor. **b.** Histological features of the metastatic colorectal cancer PDXs (I-VI); varying degrees of differentiation from well formed glands (IV, V, VI) to sheet of cells without gland formation (I, II, III). Matched PDX cell lines differ in shape: Fibroblast-like (VII), epithelial-like (IX, X) and lymphoblast-like (VIII, XI, XII) cells

In order to implement a precision medicine strategy for the treatment of metastatic CRC, a CRC precision medicine pipeline was created. We first developed patient derived models of cancer including low passage cell lines and patient derived xenografts (PDX) for patients undergoing resection of their CRC liver metastasis or primary colon at Duke University under an Institutional Review Board (IRB)- and Institutional Animal Care and Use Committee (IACUC)-approved protocol. For each patient, matching cell lines and PDXs were developed as previously described [11, 12]. CRC057, CRC119, CRC240, CRC247 and 15–496 were derived from CRC liver metastasis, and 16–159 was derived from a primary colon cancer. Among the five patients who underwent liver resection, 2 were synchronous presenters and 3 were metachronous presenters. Only 1 patient (CRC119) received neoadjuvant chemotherapy with FOLFIRI prior to resection. Patient demographics are described in Fig. 1a, and Fig. 1b shows the histological features of PDXs (I-VI) and cell lines (VII-XII).

### High-throughput drug screening in vitro and in vivo validation of cytotoxic chemotherapy agents

**Fig. 2:**
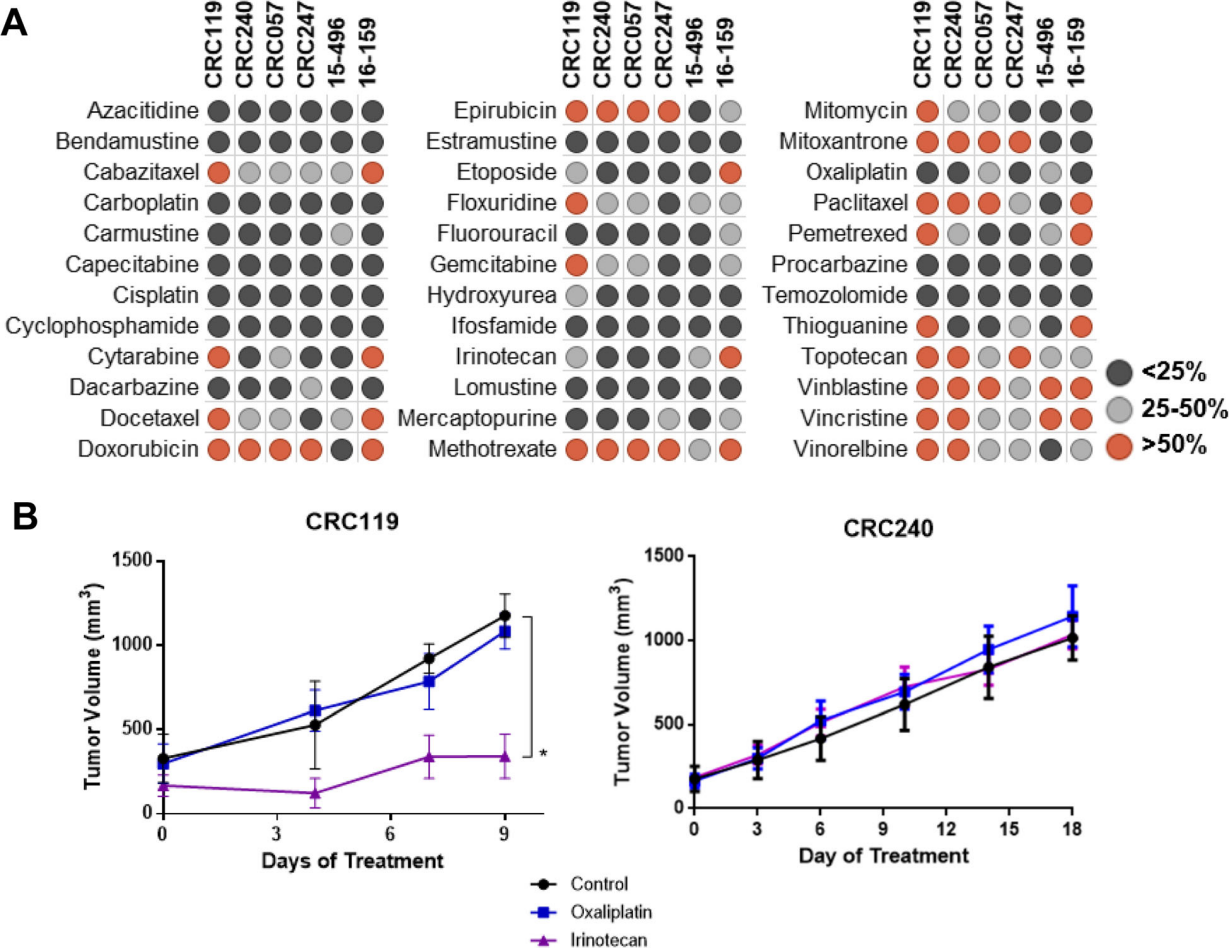
Consistency in drug sensitivity and resistance between matched patient-derived cell line and PDX tumor. **a.** High-throughput drug screening using patient-derived cell lines in vitro revealed sensitivity and resistance to a number of commonly-used cytotoxic chemotherapeutic agents (red dots > 50% killing; gray dots between 25 and 50% killing; black dots: < 25% killing). **b.** In vitro effect of standard-of-care agents (oxaliplatin and irinotecan) were validated on matched PDX tumors (2-way ANOVA: * *p* = 0.0002)

As the first step in identifying potential therapeutic targets, we performed a series of in vitro high-throughput drug screens using our patient-derived cell lines. The drug screen consisted of 119 FDA-approved small molecule inhibitors, and we first analyzed responses to commonly-used cytotoxic chemotherapeutic agents. In general, our CRC cell lines appear to be sensitive to anthracyclines (doxorubicin and epirubicin) and vinca alkaloids (vincristine and vinblastine) and resistant to platinum agents (cisplatin and carboplatin) and alkylating agents (ifosfamide and cyclophosphamide) (Fig. 2a). We next analyzed the response to standard-of-care cytotoxic agents used in the treatment of metastatic CRC, including oxaliplatin, irinotecan and 5-fluorouracil. CRC057 and 15–496 were found to be relatively sensitive to oxaliplatin, while CRC119, CRC240, CRC247 and 16–159 were found to be resistant (Fig. 2a). In contrast, CRC119 and 16–159 were relatively sensitive to irinotecan, while CRC057, CRC240, CRC247 and 15–496 were resistant (Fig. 2a).

To validate our in vitro screening results, we performed in vivo validation on matched PDX tumors using standard-of-care cytotoxic agents. Mice at 10 weeks of age (~ 25 g) were divided into 2 groups (control and treatment, consisting of 5 mice/group) and treated with oxaliplatin (10 mg/kg) and irinotecan (10 mg/kg) intraperitoneally five times a week. Consistent with our in vitro data, the CRC119 PDX tumor was sensitive to irinotecan (2-way ANOVA, *p* = 0.0002) and resistant to oxaliplatin treatment. Similarly, as predicted by our in vitro drug screen CRC240 PDX was resistant to both chemotherapeutic agents (Fig. 2b). No significant adverse events were seen. Finally, as previously described, 16–159 PDX was sensitive to irinotecan and resistant to oxaliplatin [14]. Together, these studies indicated that our screening and validation platform enabled rapid analysis of sensitivity and resistance to standard-of-care agents.

### High-throughput drug screening identifies ponatinib as a novel therapeutic target

**Fig. 3:**
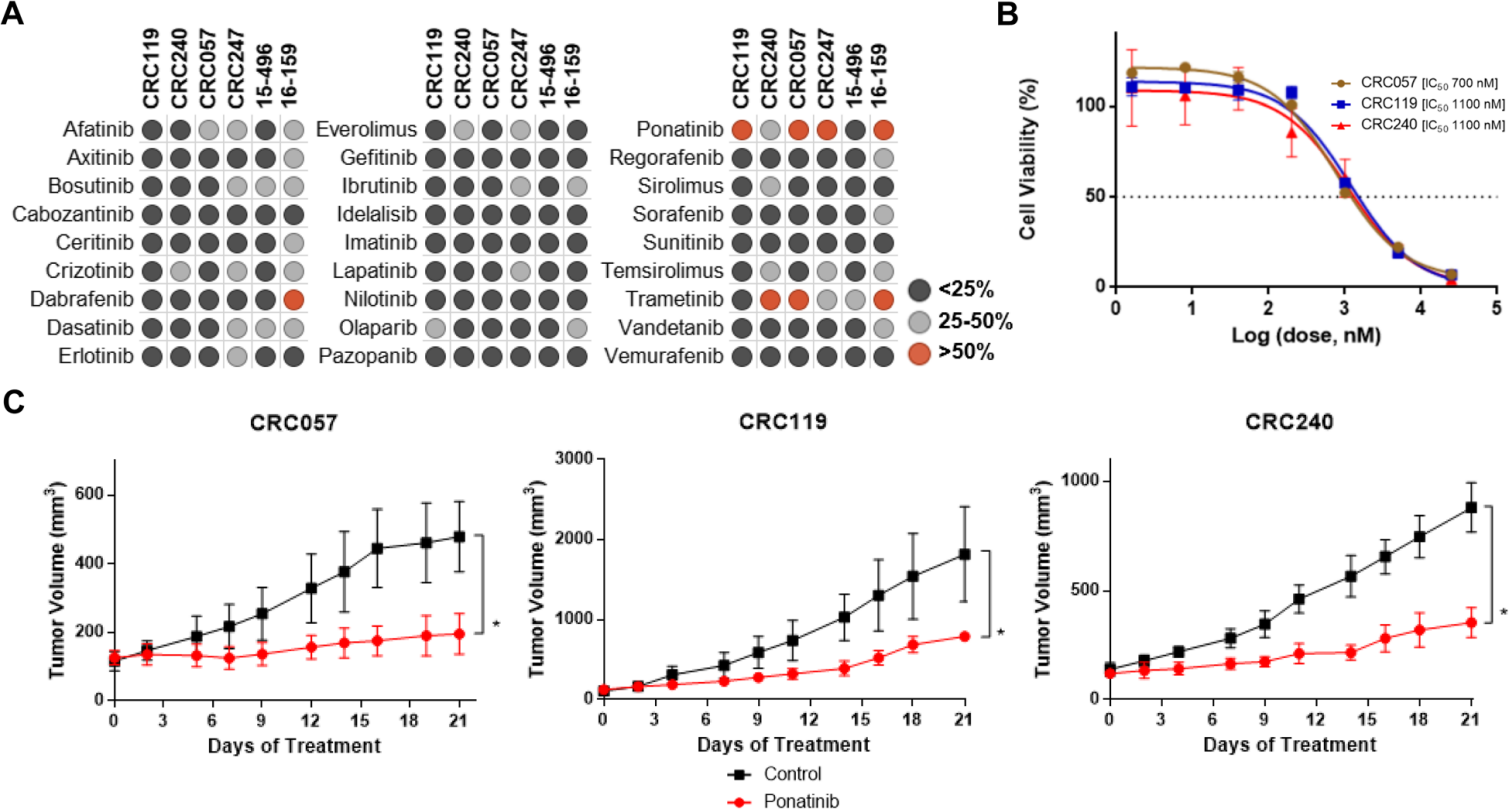
Ponatinib, a multi-kinase inhibitor, inhibits colorectal cancer cell growth in vitro and in vivo. **a**. A high-throughput drug screening identified ponatinib as a novel therapeutic target (red dots > 50% killing; gray dots between 25 and 50% killing; black dots: < 25% killing). **b.** Drug sensitivity studies were performed on CRC057, CRC119 and CRC240 cell lines to determine IC50s to ponatinib. **c.** CRC057, CRC119 and CRC240 matched PDXs were treated orally with 30 mg/kg ponatinib five times a week

Next, to identify novel targeted agents to treat metastatic CRC, we mined our drug screen data for targeted therapies for which one or more cell lines were inhibited by > 50%. Interestingly, only a limited number of targeted therapeutic agents inhibited cell growth in vitro, including dabrafenib, trametinib, and ponatinib. Among these, ponatinib inhibited growth of 4/6 cell lines at > 50% (Fig. 3a). To further characterize the effect of ponatinib in CRC, drug sensitivity studies were performed on the cell lines to determine the IC_50_ of ponatinib. The estimated IC_50_ values were 0.7 μM for CRC057, 1.1 μM for CRC119 and 1.1 μM for CRC240 (Fig. 3b). To validate the efficacy of ponatinib inhibition in vivo, we used matched PDX models of the cell lines. CRC119, CRC240 and CRC057 were injected subcutaneously in the flanks of the mice (at 10 weeks of age and ~ 25 g) as previously described [11, 12], were divided into 2 groups (control and treatment, consisting of 5 mice/group) and treated with 30 mg/kg oral ponatinib five times a week. Consistent with the in vitro results, CRC057, CRC119 and CRC240 were all found to be sensitive to ponatinib (2-way ANOVA, *P* < 0.0001) (Fig. 3c). No significant adverse events were seen. Together, these in vitro and in vivo studies indicate that using matched patient-derived cell lines and PDXs can provide a robust screening and in vivo validation platform to identify personalized therapies to treat CRC.

### Targeting ponatinib in CRC

**Fig. 4:**
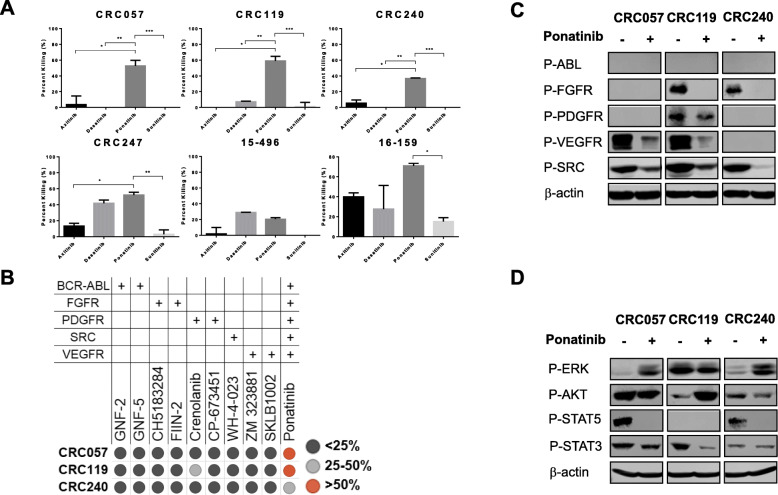
Ponatinib shows its antitumor activity by targeting multiple signaling pathways. **a**. CRC057, CRC119 and CRC240 were resistant to axitinib, sunitinib and dasatinib, suggesting that the mechanism of action of ponatinib in these three cell lines may be through inhibiting a common signaling pathway or pathways (one-way ANOVA, Tukey’s multiple comparison test: *, **, *** *p* < 0.05, the error bars represent standard deviation among replicates). **b.** Drug screening with the specific inhibitors of ponatinib targets. **c**. In vitro pre- and post-treatment western blot analysis of the intracellular tyrosine kinase domains of the ponatinib targets. **D.** In vitro pre- and post-treatment western blot analysis of the downstream signaling pathways of ponatinib’s targets

Our personalized medicine pipeline identified ponatinib as a potentially effective agent to treat CRC. Ponatinib is a multi-kinase inhibitor that targets the fibroblast growth factor receptor (FGFR), platelet derived growth factor receptor (PDGFR), vascular endothelial growth factor receptor (VEGFR), SRC, and ABL [15]. As ponatinib is a multi-kinase inhibitor, in order to identify the main target of ponatinib in our cell lines, we re-analyzed our screen data to identify other agents that target similar pathways as ponatinib. We identified three other agents, including axitinib, a VEGFR and PDGFR inhibitor; sunitinib, a PDGFR inhibitor; and dasatinib, a SRC and ABL inhibitor [16–20]. Remarkably, CRC057, CRC119 and CRC240 were all resistant to axitinib, sunitinib, and dasatinib, suggesting that the mechanism of action of ponatinib in these three cell lines might be through inhibiting a common signaling pathway or pathways (Fig. 4a).

To identify the common pathway or pathways that drive CRC growth in these patient-derived cell lines, we next screened CRC057, CRC119 and CRC240 with specific inhibitors of ponatinib’s targets, including ABL, FGFR, PDGFR, SRC and VEGFR. However, all three cell lines were found to be resistant to the specific inhibitors (Fig. 4b), suggesting that ponatinib was showing its antitumor activity by targeting multiple signaling pathways.

Pre-treatment analysis of the cell lines with p-FGFR, p-VEGF, p-PDGFR, p-SRC and p-ABL antibodies showed that all three cell lines expressed a variety of intracellular tyrosine kinase receptors (Fig. 4c). The IC50 dose of ponatinib inhibited p-FGFR activity in CRC119 and CRC240; p-VEGFR activity in CRC057 and CRC119; p-PDGFR activity in CRC 119; and p-SRC activity in all three cell lines (Fig. 4c), suggesting that different signaling pathways are implicated in determining sensitivity to ponatinib. To further verify the cell growth inhibition by ponatinib, we next focused on downstream signaling pathways of FGFR, VEGFR, PDGFR and SRC including the PI3K/AKT/mTOR, RAS/RAF/MEK/ERK, and STAT pathways. Pre- and post- treatment western blot analysis showed that STAT pathways were consistently targeted in all three cell lines. In contrast, p-AKT increased in response to ponatinib treatment, suggesting that the PI3K/AKT/mTOR pathway was activated in CRC119. Similarly, p-ERK expression increased in CRC057 and CRC240, suggesting that the RAS/RAF/MEK/ERK pathways were activated in response to ponatinib treatment (Fig. 4d).

### Determining the molecular predictor of sensitivity to ponatinib

To better understand the potential underlying genetic determinants of our patient-derived models of cancer to ponatinib, we performed RNA-Seq on the cell lines. Specifically, we found two mutations in the FGFR1 open reading frame, three mutations in the FGFR2 open reading frame and three mutations in the FGFR4 open reading frame in our six cell lines. We observed A254V and S429fs mutations in FGFR1 in CRC119 and 16–159, respectively. Similarly, we found P470L and W76R mutations in FGFR2 in CRC119 and CRC240, respectively, and M71T mutation in FGFR2 in 16–159. None of the cell lines were found to have mutation in FGFR3. In all six cell lines, we observed a P136L mutation in FGFR4. G388R mutation was also observed in FGFR4 in the CRC057, CRC240 and 16–159 cell lines. In addition to this, V10I mutation was found in FGFR4 in only the 16–159 cell line (Table 1). A complete list of all mutations found in FGFR is listed in Table S1. While all lines had synonymous, intronic, or upstream/downstream mutations in SRC, CRC119 also had mutations in the 5′ and 3′ untranslated regions, and both CRC119 and CRC247 had three mutations within the 3′ untranslated region as well as a 5′ splice site mutation within exon 2 of SRC. No mutations were found in VEGFR.

**Table 1:** Nonsynonymous exonic single nucleotide polymorphisms of the six metastatic colorectal cancer matched cell lines

Sample ID	FGFR1	FGFR2	FGFR2	FGFR4
CRC057		M71T		P136L, G388R
CRC119	A254V	P470L		P136L
CRC240		W76R		P136L, G388R
CRC247				P136L
15–496				P136L
16–159	S429fs	M71T		V10I, P136L, G388R

## Discussion

Patient derived models of cancer are accepted as efficient tools for the development of the cancer therapeutics [21]. Specifically, morphological and molecular mimicry between these models and the original patient tumors facilitate the evaluation of anticancer drug responses and resistance [22]. Recently, high-throughput drug screens of patient-derived organoids followed by validation of drug candidates in patient derived xenograft (PDX) models has been coupled with genomic analysis to develop personalized medicine platforms in various types of cancer [10]. Specifically, in colorectal cancer (CRC), matched PDX and cell line platform has been used as a preclinicaltool for functional gene validation and proof-of-concept studies to identify novel druggable vulnerabilities [23]. Additional studies such as using tumor organoid cultures from CRC specimens to perform drug screens [7] or patient derived xenografts of CRC to predict drug response [8] have paved the way for the use these patient derived models of cancer to identify and develop new therapeutics.

In this study, we have developed our own precision medicine strategy for patients with metastatic CRC using matched cell lines and PDX platform coupled with high throughput drug screens and genomic analyses to identify novel targets and potential predictive biomarkers. The similar responses of the matched cell lines and PDX tumors to standard-of-care CRC treatment agents, including oxaliplatin and irinotecan, suggest that our strategy is a reliable means to identify effective therapies. Interesting enough, our one patient who received neoadjuvant thearpy prior to resection of their cancer (CRC119) was found to be responsive to an irinotecan therapy and simlary her cell and PDX were also found to be sensitive to irinotecan (Fig. 2) In addition to our evaluation of standard-of-care agents, our high-throughput drug screening using matched cell lines allowed us to discover several pathways of interest, including FGFR, PDGFR, and VEGFR, all of which may contribute to CRC growth. Ponatinib, a multi-kinase inhibitor of these pathways, significantly inhibited cell growth in vitro and PDX tumor growth in vivo in our CRC models. Ponatinib was initially designed to inhibit BCR-ABL [15], and provided to the patients who were resistant to dasatinib or nilotinib treatment. In addition to its effective inhibition of both wild type and several mutant forms of BCR-ABL kinases [24], further studies have demonstrated ponatinib’s ability to target several other tyrosine kinases [25]. However, our screening data suggested that there was no antitumor activity with the single kinase inhibitors of the ponatinib’s other targets. This can be explained by 1) synergistic effect from co-targeting these receptors as Lee et al. previously reported effective tumor inhibition in in vivo colon cancer models with CHIR-258, which is EGFR, FGFR, PDGFR and VEGFR inhibitor [26], 2) overlapping downstream pathways of these receptors, that might allow cancer cells to develop resistance mechanisms using alternative receptor tyrosine kinases. Ellis et al. points out this resistance mechanisms and specifically compensatory activation of FGFR pathway after VEGFR inhibition [27]. Consistent with these studies, we also demonstrated that co-targeting these receptors can effectively inhibited tumorigenesis. Interestingly, we observed downstream activation of the RAS/RAF/MEK/ERK pathway in CRC240 and CRC057 and the PI3K/AKT/mTOR pathway in CRC119 after ponatinib treatment, which may indicate these pathways as potential resistance mechanisms.

Deregulation of the FGFR signaling pathway plays an important role in carcinogenesis [28]. Genomic alterations in the FGFR genes that enhance FGFR signaling are mediated by either receptor amplification, mutations or chromosomal translocation [29]. Specifically, FGFR amplification has been found in lung and breast cancer, and response to FGFR inhibition has been found in amplified FGFR tumors [30–32]. In addition to somatic activating mutations, germline single-nucleotide polymorphisms (SNPs) in FGFR have been found to activate the FGFR pathway [33]. Finally, activating gene fusions of FGFR have been discovered in a number of different cancers [34, 35]. In colorectal cancer, genomic alterations in FGFR such as gene amplifications [36] are not as common as fusion in FGFR3 [37] or gene copy number gain in FGFR1 [38]. Along these lines, we used RNA-Seq data to potential chromosomal translocations or mutations to identify predictors of response. While no fusions were found (Supplementary Figure 1), mutations were found in FGFR1, 2 and 4, with the most common mutation being P136L in FGFR4 in all six samples. Although these are potentially interesting findings, we realize that this is a limited analysis and further studies will need to be performed to validate these findings, but the incoproation of genomic profiling to complement functional studies remain a critical component of any precision medicine pipeline.

Simarily, we do realize the limitations of our current precisioin medicine pipeline. As this was our intial proof of concept developoment of our pipeline, our drug screen contained only 119 drugs and the majority of the targeted agents in our screen targeted multiple pathways suggesting that in our screen, combinatorial therapy may be critical to find the optimal therapy in CRC. In addition, our screen which used cell lines limits our in vitro studies that involves anticancer compounds that target the microenvironment. Therefore, this limitation may cause underpredicted in vitro cell line response to these compounds, as in CRC240, which was found to be moderately sensitive to ponatinib in vitro, but quite sensitive in vivo. Despite these challenges, in vitro cell line models have still been widely used for initial pharmacogenomic studies as they allow for simple and low cost biological research but future work will determine if using patient derived organoids which overcome the challenges of cell lines can be used in our precision medicine pipeline.

The development of precision medicine strategies for cancer faces numerous challenges, including accessing patient samples, establishing reliable models for testing, and the genetic and non-genetic diversity inherent within the ever-evolving cancer. Here we propose a pipeline and workflow to address several of these challenges. By establishing patient-matched cell lines and PDXs we are able to leverage the speed and flexibility of in vitro systems while simultaneously providing a robust system for in vivo validations that takes into account, at least in part, tumor heterogeneity and contributions of the tumor microenvironment. Indeed, we have previously shown that PDXs faithfully recapitulate patient tumor histology and preserve tumor-associated stroma and coupled with high-throughput screens and genomics, this pipeline represents a useful paradigm to identify and validate new treatments for CRC that can be expanded to other solid tumors.

## Conclusions

Our current work has provided a framework for a precision medicine approach to identify new treatments for patients with metastatic CRC (**Supplementary Figure****2**). Future studies will be focused on 1) determining if combining next generation sequencing with drug screening and validation using patient-derived models of cancer is informative and useful to guide patient care after standard-of-care therapy is completed, either using a clinical trial or with off-label use; and 2) comparing the efficacy of treatment of cell lines and PDXs to patient clinical outcomes with respect to overall tumor response and duration of response.

## Supplementary information

**Additional file 1: Supplementary Figure 1.** RNA-Seq data to identify fusion and mutations in FGFR1–4. **Supplementary Figure 2.** Precision Medicine Strategy for Metastatic Colorectal Cancer. **Supplementary Figure 3.** Uncropped western blots showing all the bands with the molecular weight markers.

**Additional file 2: Supplementary Table 1.** Mutations in FGFR1–4

## Data Availability

The datasets used and/or analysed during the current study are available from the corresponding author on reasonable request.

## Abbreviations

CLA: Cell line authentication
CRC: Colorectal cancer
FBS: Fetal bovine serum
FGFR: Fibroblast growth factor receptor
HRP: Horse radish peroxidase
IACUC: Institutional animal care and use committee
IRB: Institutional review board
PDGFR: Platelet derived growth factor receptor
PDX: Patient-derived xenografts
RIPA: Radioimmunoprecipitation assay
RNA-Seq: RNA sequencing
STR: Short tandem repeat
VEGFR: Vascular endothelial growth factor receptor
