# Supplementary material for: Development of a precision medicine pipeline to identify personalized treatments for colorectal cancer
Source: BMC Cancer. 2020 Jun 24;20:592. doi: 10.1186/s12885-020-07090-y (PMC7313200; doi:10.1186/s12885-020-07090-y)
Supplement: Supplementary file 1 — Additional file 1: Supplementary Figure 1. RNA-Seq data to identify fusion and mutations in FGFR1–4. Supplementary Figure 2. Precision Medicine Strategy for Metastatic Colorectal Cancer. Supplementary Figure 3. Uncropped western blots showing all the bands with the molecular weight markers. [file 12885_2020_7090_MOESM1_ESM.pdf]

Supplementary Figure 1

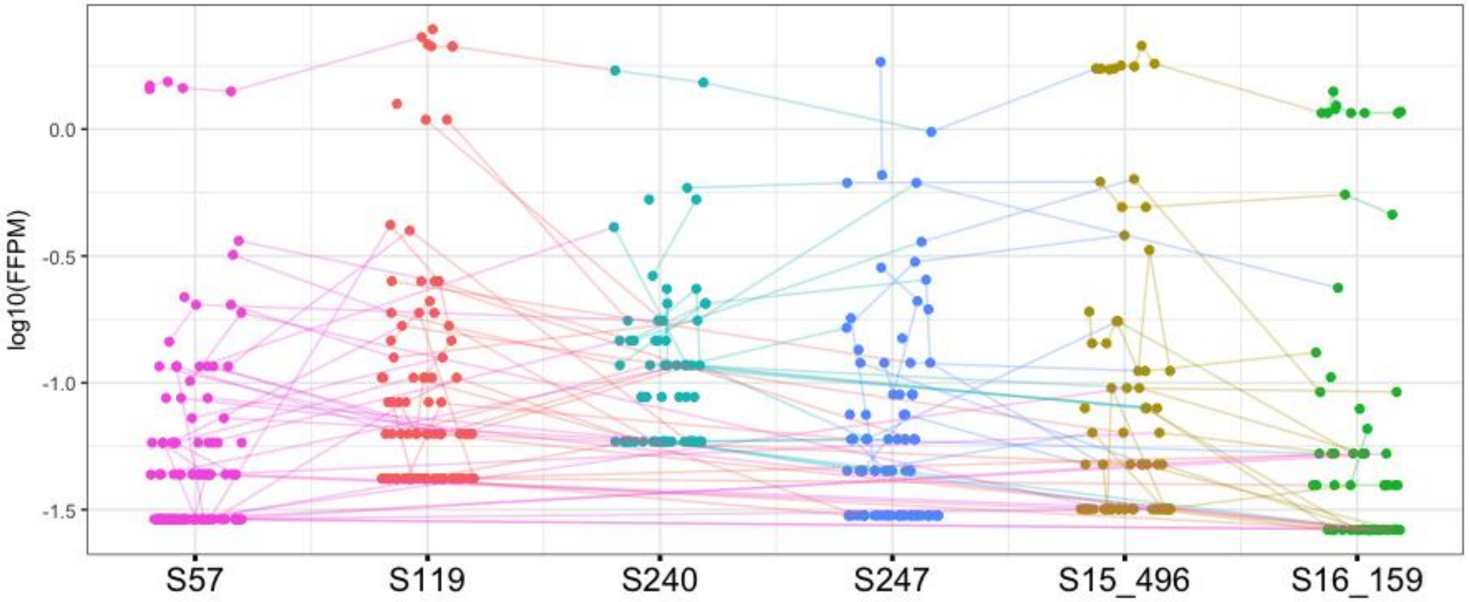

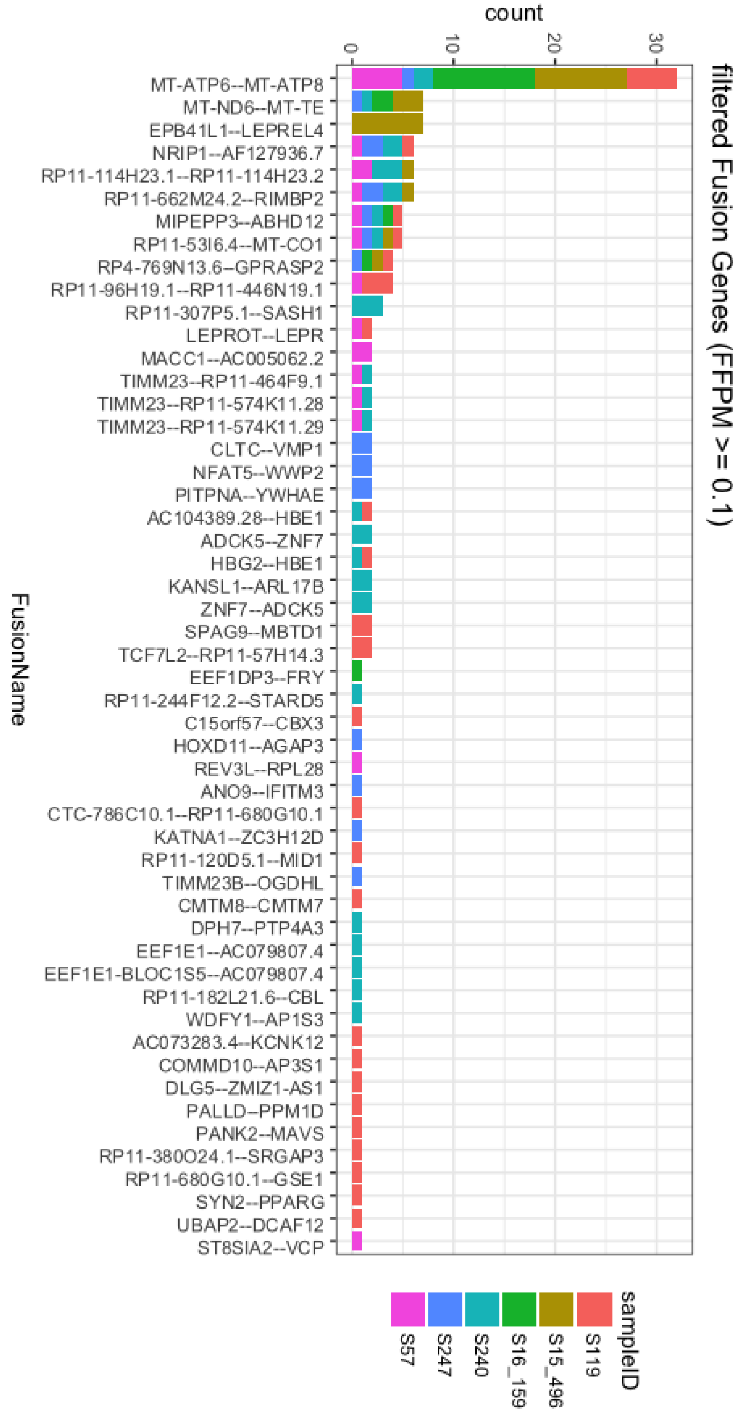

Supplementary Figure 2

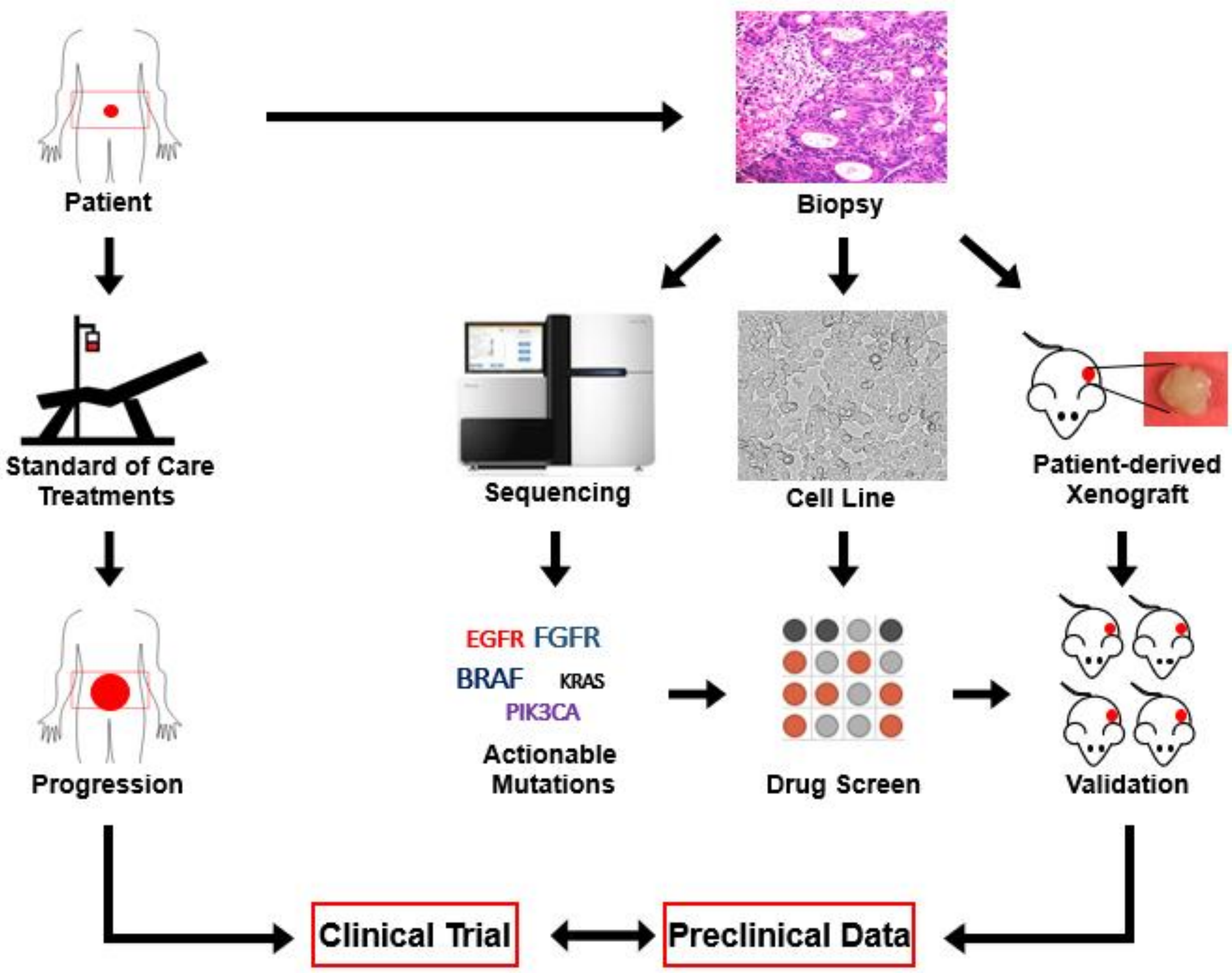

Supplementary Figure 3

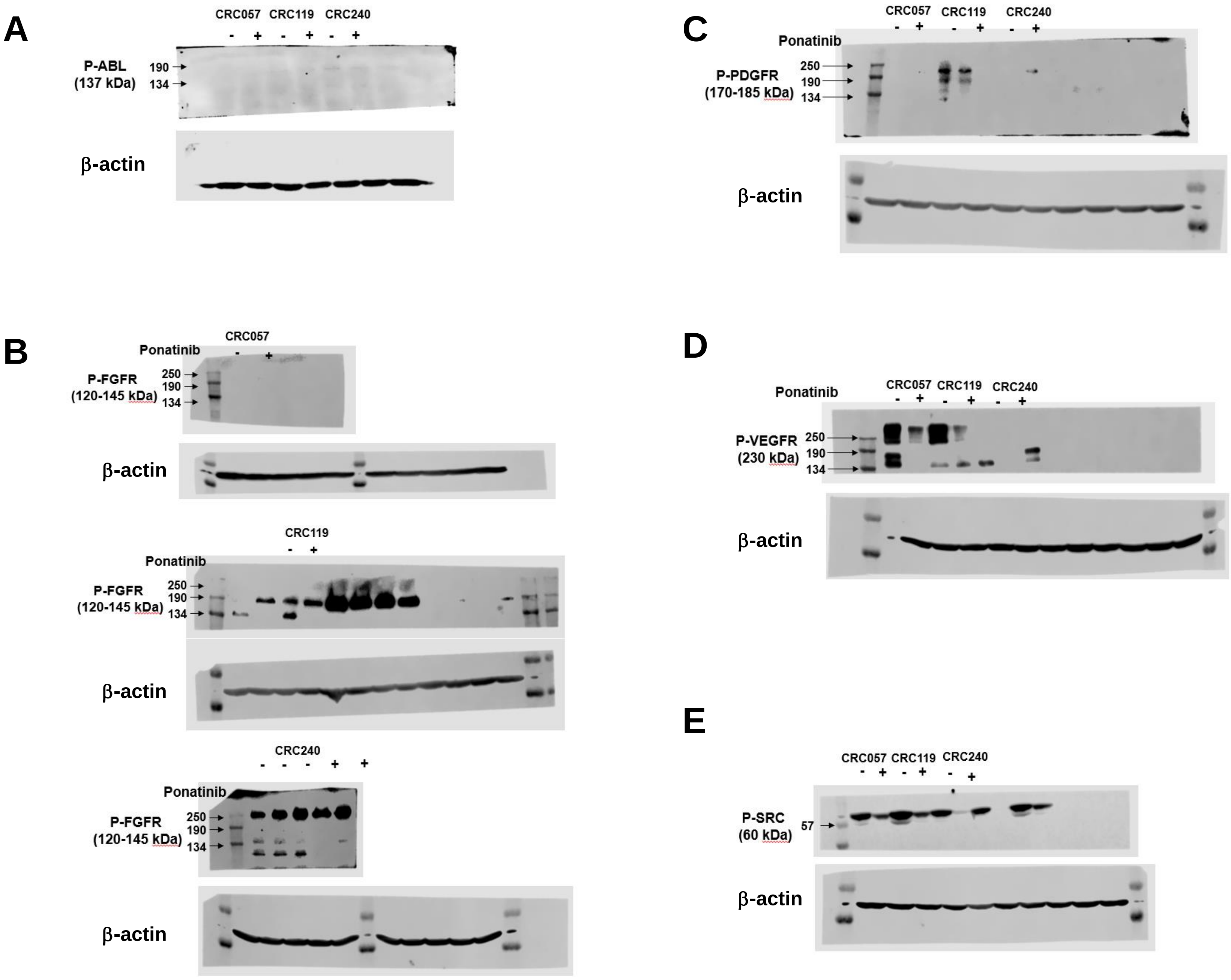

A. Uncropped blot of p-Abl of Figure 4C
B. Uncropped blot of p-FGFR of Figure 4C
C. Uncropped blot of p-PDGFR of Figure 4C
D. Uncropped blot of p-VEGFR of Figure 4C
E. Uncropped blot of p-SRC of Figure 4C

Supplementary Figure 4

A

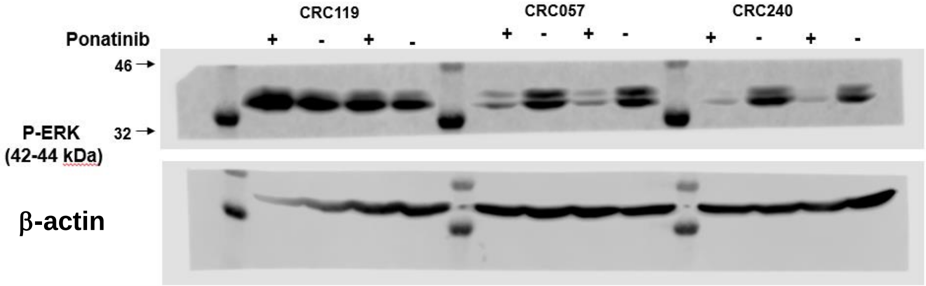

B

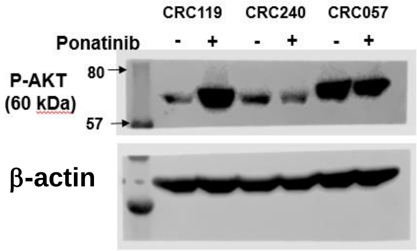

C

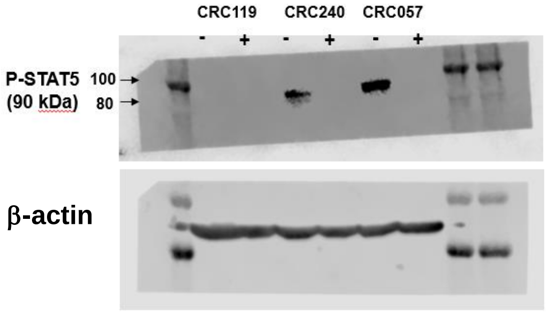

D

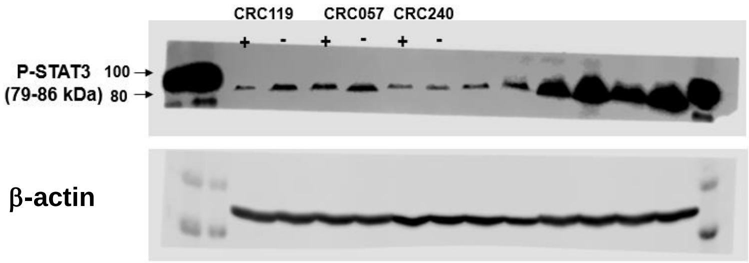

A. Uncropped blot of p-ERK of Figure 4D
B. Uncropped blot of p-AKT of Figure 4D
C. Uncropped blot of p-STAT5 of Figure 4D
D. Uncropped blot of p-STAT3 of Figure 4D
